# Global transcriptome profiles provide insights into muscle cell development and differentiation on microstructured marine biopolymer scaffolds for cultured meat production

**DOI:** 10.1038/s41598-024-61458-9

**Published:** 2024-05-13

**Authors:** Dragica Bezjak, Nicole Orellana, Guillermo Valdivia, Cristian A. Acevedo, Jorge H. Valdes

**Affiliations:** 1 Centro de Biotecnología, Universidad Técnica Federico Santa María, Avenida España 1680, Valparaíso, Chile; 2 Center for Bioinformatics and Integrative Biology, Facultad de Ciencias de la Vida, Universidad Andrés Bello, Avenida República 239, Santiago, Chile; 3 Departamento de Física, Universidad Técnica Federico Santa María, Avenida España 1680, Valparaiso, Chile; 4 Centro Científico Tecnológico de Valparaíso (CCTVal), Universidad Técnica Federico Santa María, Avenida España 1680, Valparaiso, Chile

**Keywords:** Cultured meat production, Transcriptomics, Edible scaffolds, Network modeling, Computational biology and bioinformatics, Functional clustering, Gene ontology, Gene regulatory networks, Bioinspired materials, Biomaterials - cells, Biomaterials, Functional genomics, Biotechnology, Sequencing, RNA sequencing

## Abstract

Biomaterial scaffolds play a pivotal role in the advancement of cultured meat technology, facilitating essential processes like cell attachment, growth, specialization, and alignment. Currently, there exists limited knowledge concerning the creation of consumable scaffolds tailored for cultured meat applications. This investigation aimed to produce edible scaffolds featuring both smooth and patterned surfaces, utilizing biomaterials such as salmon gelatin, alginate, agarose and glycerol, pertinent to cultured meat and adhering to food safety protocols. The primary objective of this research was to uncover variations in transcriptomes profiles between flat and microstructured edible scaffolds fabricated from marine-derived biopolymers, leveraging high-throughput sequencing techniques. Expression analysis revealed noteworthy disparities in transcriptome profiles when comparing the flat and microstructured scaffold configurations against a control condition. Employing gene functional enrichment analysis for the microstructured versus flat scaffold conditions yielded substantial enrichment ratios, highlighting pertinent gene modules linked to the development of skeletal muscle. Notable functional aspects included filament sliding, muscle contraction, and the organization of sarcomeres. By shedding light on these intricate processes, this study offers insights into the fundamental mechanisms underpinning the generation of muscle-specific cultured meat.

## Introduction

Over the last 50 years, global meat production has increased steadily as the demand for meat consumption increases; this growth will continue in the coming decades^1^. However, this is unsustainable because conventional meat production is problematic. According to the Food and Agriculture Organization (FAO), the total emissions from global livestock contribute to 14.5% of all greenhouse gas emissions and use 8% of global freshwater^2^. To meet the growing demand for animal products, grasslands and forests have been cleared worldwide to raise livestock^3^. Health concerns such as nutrition-related diseases and food-borne illnesses arise from conventional meat production due to intensive factory farming and poor animal welfare conditions^4^. They also contribute to disease outbreaks such as bovine spongiform encephalopathy and swine flu, promoting the use of antibiotics in animal farming for increased feed efficiency leading to antimicrobial-resistant pathogens, and threatening new healthcare crises^5^.

Moreover, ethics regarding the raising of livestock and the slaughtering of animals have also been questioned. Animal welfare is often ignored in factory farms to keep up with production efficiency^6^. Apart from the poor living conditions, the animal feeding time is systematic and frequent, forcing them into a desirable size or weight before slaughtering^7^.

Cultured meat is viewed as one promising alternative since healthy muscle cells are used for food production without compromising nutritional profile and slaughtering animals^8^. Meat analogs are alternatives to produce sustainable foods that replace traditional animal protein sources^9^. Despite advancements in tissue engineering and 3D tissue culture, it is challenging to replicate meat tissue due to its complex arrangement of different cells, extracellular matrix, proteins, nutrients, and growth factors^10^.

The large-scale production of cultured meat remains economically impractical due to its foundation in knowledge primarily derived from medical applications^11^. However, advancing our understanding of muscle tissue engineering for culinary purposes is imperative, particularly in the enhancement of attributes like color and other sensory properties^12^. To transform cultured meat into a financially viable food source, it is imperative to delve into three pivotal aspects: (i) the creation of animal-free growth media, (ii) the utilization of edible scaffolding, and (iii) the deployment of suitable bioreactors. Generating cultured meat usually involves cultivating myoblasts in suspension or on a scaffold within a serum-free culture medium inside a bioreactor^13^.

A scaffold is a matrix (soft material) where the anchorage-dependent cells (e.g., muscle cells) can adhere, remain viable, proliferate, and differentiate^14^. In addition, using mammalian components in the scaffold should be avoided to effectively reduce the slaughter of bovines^15^. Research on scaffold generation for cultured meat is still scarce, and it is mainly focused on developing microstructures to align muscle tissue formation^15,16^. Xiang et al.^17^ recently manufactured edible scaffolds for use in cultured meat using different biomaterials and concluded that those based on proteins show better results in the adhesion, proliferation, and differentiation of muscle cells. Furthermore, Zhu et al.^18^ optimized a culture medium to accelerate proliferation, maintain muscle cell differentiation, and improved a mold to develop microstructure scaffolds to allow cell alignment.

In prior work, we developed an optimized formulation based on non-mammalian components to make edible scaffolds suitable for myoblast culture^14^. The scaffold was formulated with a mix of three marine biopolymers (salmon gelatin, alginate, and agarose), where salmon gelatin works as a functional macromolecule containing RGD (arginine-glycine-aspartic acid) sequences that promote cell adhesion and proliferation^19,20^. Salmon gelatin has advantages over mammalian gelatin, such as lower risk of disease transmission and greater acceptance from diverse countries and their cultures^21^. In addition, it has differences in the number of amino acids such as proline, hydroxyproline, threonine, and serine (which depends on the type of fish from which the gelatin is obtained), resulting in changes in the physical–chemical properties of the gelatin, such as gelling temperature, viscoelasticity, and gel strength^21,22^. Despite the contribution of salmon gelatin to scaffold generation, it is well known that the use of any animal-derived components is not aligned with the goals of cultured meat^4^. However, to promote the transition from medical-like to culinary approaches for in vitro meat production, the use of non-mammalian discarded material, such as salmon skin, provides an economically viable step for the continuous development of novel tailored scaffolds for cultivated meat.

Alginate, an anionic polysaccharide composed of β-D-mannuronic acid (M) and α-L-guluronic acid (G), has been used in the food industry as it can form gels when crosslinked with di or trivalent ions or with positively charged macromolecules^23^. Agarose is a linear polysaccharide composed of 1,3-linked-β-D-galactose and 1,4-linked 3,6-anhydrous-α-L-galactose repeating units alternately, has great gelling power, is thermoreversible, and has good film-forming ability^24^.

The use of transcriptomics has provided a complementary approach to exploring the complete gene expression landscapes in biological and food systems^25^. Recently, unbiased sequencing methodologies have been available for genome-wide high-throughput transcriptomics. It has been applied to investigate skeletal muscle transcription profiles of cattle to uncover the regulatory mechanisms affecting muscle development^26^. High-throughput RNA sequencing has been used to investigate bovine muscle cells during myogenic differentiation^27^, to identify cytoskeletal structural genes as markers for meat quality in beef^28^ and to characterize meat obtained from Chinese Jinjiang yellow cattle during early post-mortem^29^. Moreover, a study was developed by Denes et al.^30^, in which they used micromolded gelatin hydrogels and evaluated myotube maturation using RNA sequencing, where they confirmed that the microstructure positively affects the formation of sarcomeres. Recently, single cell RNA sequencing was used to characterize and identify cell populations in cultured meat production^31^. However, our work aims to complement the contributions described previously, since a comprehensive transcriptional assessment of cultured meat using microstructured scaffolds has not been performed.

In this study, we fabricated flat and microstructured scaffolds using edible marine biopolymers to investigate cellular and transcriptional reactions to distinct scaffold topographies. Ultimately, our research uncovers disparities in gene expression and employs in silico modeling to unveil protein interaction networks associated with genes that play pivotal roles in the structural arrangement, differentiation, and muscular functionality of a cultured meat paradigm.

## Materials and methods

### Preparation of edible flat and microstructured scaffolds for cultured meat production

**Figure 1:**
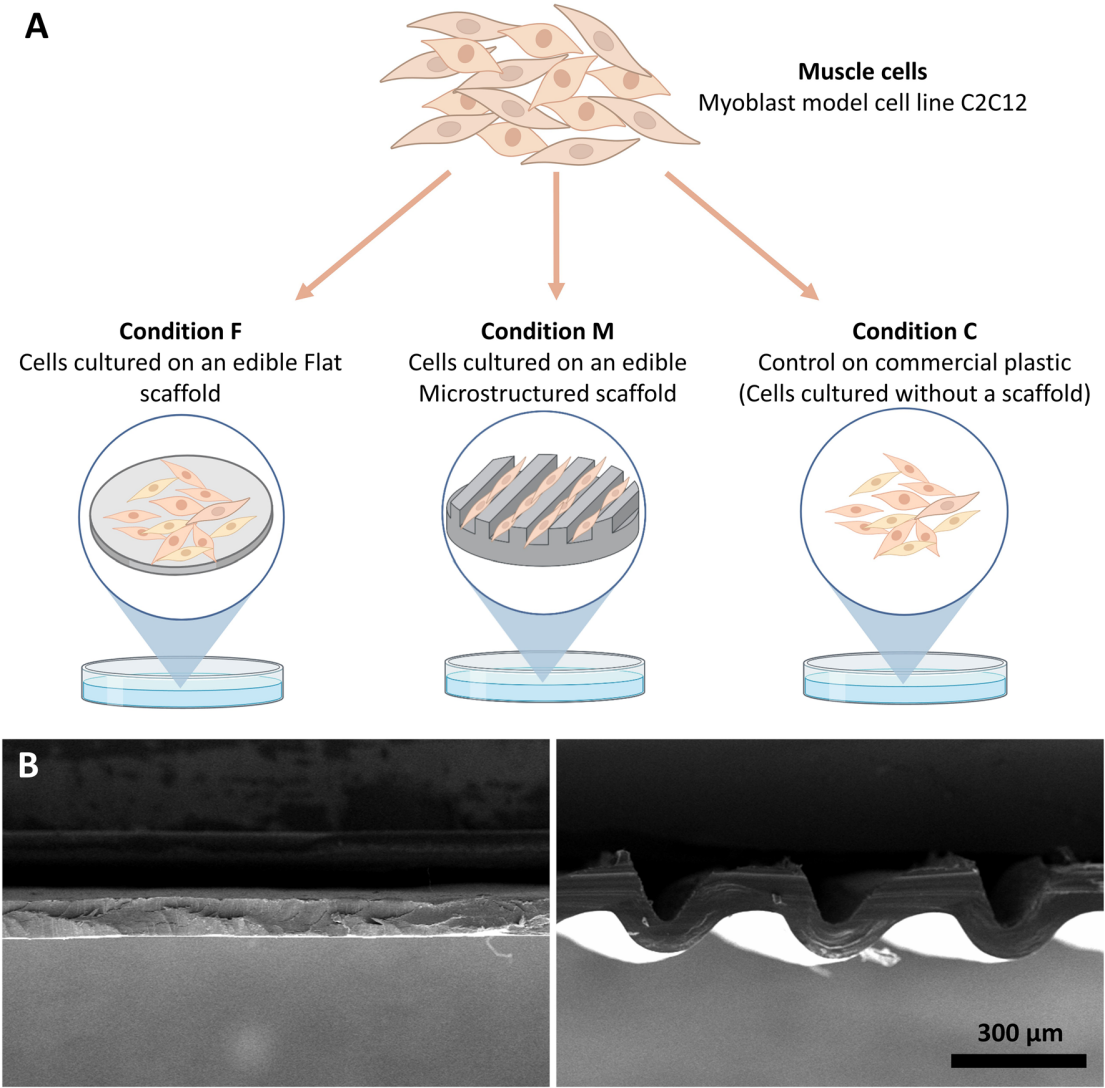
Experimental design. (**A**) Schematic representation of the three experimental conditions used in this experiment: Flat (F) scaffold, Microstructured (M) scaffold, and plastic Control (C). (**B**) SEM photographs of flat (left) and microstructured (right) scaffolds. Magnification 150×, scale bar 300 µm.

Flat and microstructured edible scaffolds were prepared using cold casting into flat and microstructured molds. Scaffolds were made using non-mammalian ingredients according to the criteria of not slaughtering bovines to make cultured meat, using our previously reported method^15,32,33^. Microstructured molds were fabricated by engraving parallel microchannels onto an acrylic plate using a laser cutter, resulting structures with approximately height of 300 µm and ridge width of 70 µm, as described by^15,32^. Since mold produces a negative shape on the final scaffold, the mold design was tailor-made to obtain bundle-like organized muscle fibers on the scaffold's surface (Fig. 1A).

The solution was prepared by combining four edible components with well-defined properties related to bioactivity (salmon gelatin 1.2%), crosslinking (sodium alginate 1.2%), gelling (agarose 0.2%), and plasticizing (glycerol 1.0%), in water as solvent. The solution was prepared at 50 °C with gentle agitation for 4 h and then poured into the molds setting the volume to obtain 4 mm of height. The molds were kept for 3 days at 10 °C to allow water evaporation, obtaining low moisture films. Before cell seeding, the films were soaked in CaCl_2_ solution (70 mM) for 1 h to crosslink the alginate fraction and obtain a non-water-soluble material. The imaging of the scaffolds was carried out using scanning electron microscopy (SEM) with a Carl Zeiss SEM (EVO MA 10, Germany) (Fig. 1B).

### Cell culture and cultured meat production

The myoblast cell line C2C12 (European Collection of Cell Cultures, ECACC) was used as a muscle cell model, as described by Orellana et al.^32^. Briefly, myoblasts were seeded onto the scaffolds, placed in a 12-well plate, at a density of 2.5 × 10^5^ cells/cm^2^. As a control, we used commercial cell culture plastic. For both the scaffolds and control we used 12-well culture plates (Falcon, Germany, Cat. N°: AZ1035C353043). Myoblasts proliferated for 3 days under standard conditions (37 °C and 5%CO_2_) using proliferation medium: DMEM high glucose with L-glutamine (2 mM) (Gibco, Life Technologies, USA) and 10% fetal bovine serum (Biologicals Industries, Israel). Then, cells were differentiated for 7 days using differentiation medium: DMEM high glucose with L-glutamine (2 mM) and 2% horse serum (Gibco, Life Technologies, USA). For more detail, see^15,32,33^.

### Myofiber analysis

Cell morphology was analyzed with cells seeded on the scaffolds (microstructured and flat) and control (commercial plastic plate) (n = 3) at a density of 2.5 × 10^5^ cells/cm^2^ using standard fluorescence techniques in an inverted microscope (Nikon, Eclipse TS2FL, Japan), staining the cells with rhodamine-phalloidin (1:200; R415; Invitrogen, Thermo Fisher Scientific, USA) and Hoechst 33,342 (1:10,000; H1399; Invitrogen, Thermo Fisher Scientific, USA)^32^. Myofiber identification was performed using the method proposed by Acevedo et al.^15^, by immunofluorescence stain of anti-myosin heavy chain (1:500; sc-376157; Santa Cruz Biotechnology, Inc). Myofiber diameter and distribution were analyzed using ImageJ.

### RNA extraction

Cells were lysed in culture plates by adding 1 mL of TRIzol Reagent (Invitrogen, USA) per sample, detached using swabs, and further homogenized by gentle pipetting. Then the RNA was purified using the RNeasy Mini Kit (Qiagen, Germany) and finally reconstituted in nuclease-free water. The RNA quantity and quality was assessed using Bioanalyzer 2100 (Agilent Technologies, USA) and Quant-iT Picogreen dsDNA assay kit (Thermo Fisher Scientific, USA) respectively.

### Library preparation and transcriptome sequencing

Three biological replicates from each condition (control, flat, and microstructured scaffolds) were used for global expression analysis. Two micrograms of total RNA with RNA Integrity Number (RIN) > 8 were used for library preparation using a TruSeq Stranded mRNA Kit with 11 cycles of PCR amplification according to the manufacturer’s recommendation (Illumina, USA). Adaptor-tagged DNA libraries were sequenced for 150 bp paired-end reads using the Illumina HiSeq SBS Kit v4 in the high-output mode according to the manufacturer’s recommendation for the Illumina HiSeq 2500 instrument at the sequencing facility of Genoma Mayor, Santiago de Chile. The sequencing data is available at the NCBI SRA public repository with BioProject accession number PRJNA882114.

### Read mapping and data analysis

Clean reads were acquired from raw reads after discarding adapter sequences from the Illumina TruSeq kit, removing low-quality, and filtering the rRNA using Trimmomatic v0.39^34^ with parameters ILLUMINACLIP:TruSeq3-PE-2.fa:2:30:10 LEADING:3 TRAILING:3 SLIDINGWINDOW:4:15 MINLEN:36. Clean reads were mapped to the GRCm39 mouse genome obtained from Ensembl using SOAP2 (parameter: m default is 5)^35^. Differentially expressed genes (DEGs) were identified through pairwise comparisons by using EdgeR (Empirical analysis of Digital Gene Expression in R)^36^ using default parameters. Gene expression was performed by TPM (transcripts per million mapped reads) method^37^. DEGs were measured according to “The significance of digital gene expression profiles”^38^. The false discovery rate (FDR) ≤ 0.05 and |log twofold change|≥ 1.5 was used as a threshold to identify DEGs. A Gene Ontology (GO)^39,40^ analysis was performed to obtain information on biological processes, molecular functions, and cellular components, by comparing the DEGs with the GO database by the Gene Ontology Consortium using AmiGO^41^.

All DEGs were mapped to GO terms, following calculated gene numbers in every term; finally, the significantly enriched GO terms were found in DEGs^42^. To identify similarities and differences in the transcriptome of the different samples, principal component analysis (PCA) and hierarchical clustering analysis (HCA) were performed using the R package. Additionally, gene associations and protein interaction networks were performed using String (https://string-db.org/) with network type Full String Network, and a minimum required interaction score of 0.700 and Cytoscape (https://cytoscape.org/) for visualization of the network with the Organic Layout option.

## Results and discussion

### Microstructured scaffolds allow cell alignment and formation of bundle-like structure

Cell alignment gives the muscle its unique structure as it allows cells to fuse and form elongated multinucleated myofibers, which are organized into bundles forming fascicles and the muscle itself^43^. To promote in vivo-like cell development is imperative to find an effective way to align the cells to obtain organized myofibers. In this study, C2C12 muscle cells were compared growing on microstructured scaffolds, flat scaffolds, and plastic as a control (Fig. 1).

**Figure 2:**
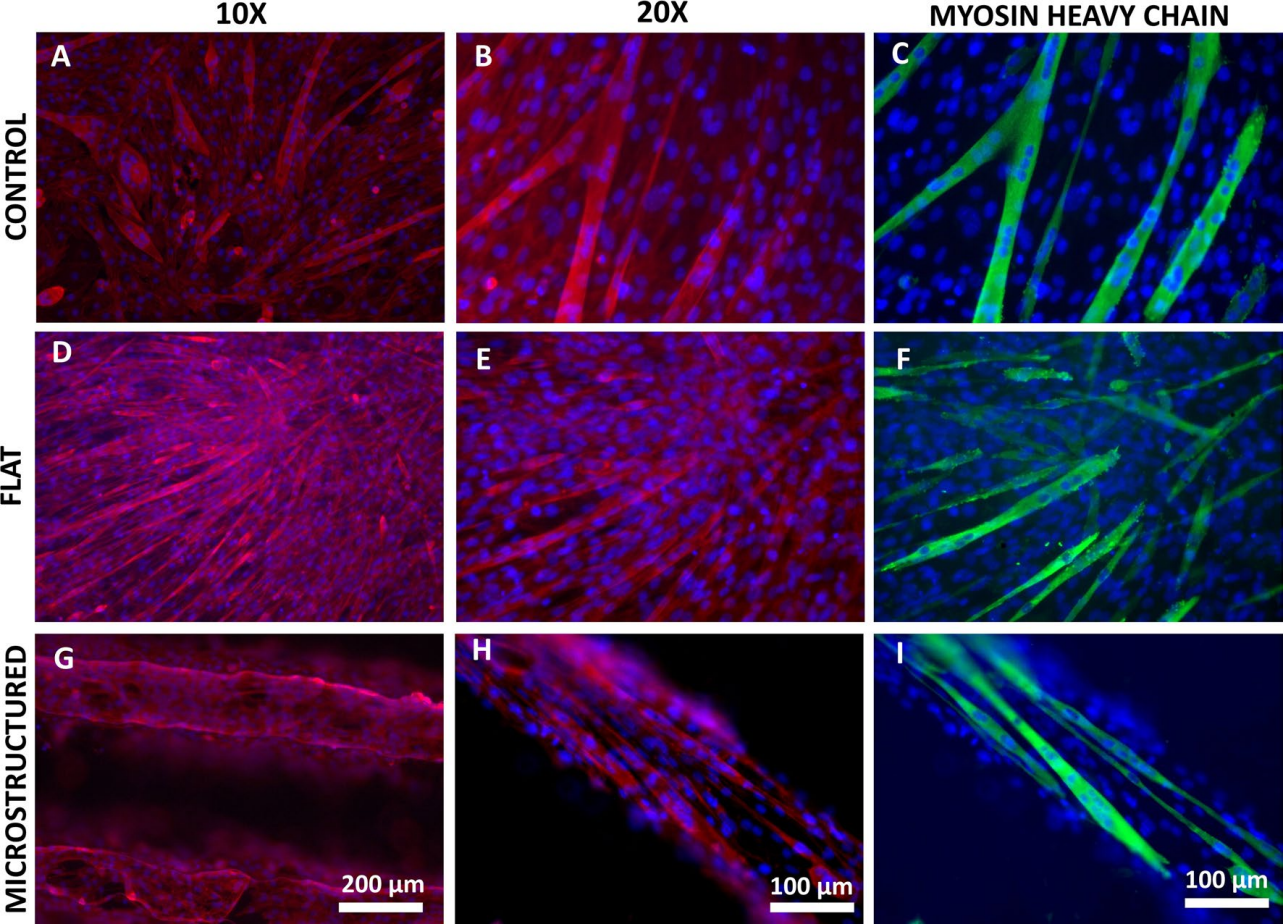
Myofiber identification of cells growing in different conditions after 7 days of differentiation in control (**A**–**C**), flat scaffold (**D**–**F**) and microstructured scaffold (**G**–**I**). Myosin heavy chain is shown in green, in red shows actin stained with Rhodamine-Phalloidin, and Hoechst-stained nuclei are shown in blue.

As shown in Fig. 2, myofiber formation occurs in all conditions (identified by myosin heavy chain stain in green), however, they differ in their organization and diameter. Cells growing on plastic tend to differentiate and grow without a common orientation (Fig. 2A–C), for which there is no myofiber bundle organization, which leads to other unwanted phenomena such as myofiber ramification, which is not typically observed in vivo (Fig. 2B–C)^30^. When cells grow on a flat scaffold, they are densely grouped and myofibers have no clear common orientation (Fig. 2D–F). Only when cells grow on a microstructured scaffold cell orientation is observed (Fig. S1), where cells are aligned with each other, following the microchannel direction. This prevents ramifications and promotes bundle-like structure organization (Fig. 2G–I).

Regarding myofibers diameter, reports in mammals vary according to the age of the animal, with a range of 10 to 100 µm, being larger as they grow^44^. Our results show that larger diameter myofibers were found in controls, with an average of 22.98 µm (± 7.44), and not much difference was found in microstructured and flat scaffolds, with average sizes of 15.25 (± 4.81) and 14.72 (± 4.56) µm, respectively (see Fig. S2).

We can observe that when cells grow on a microstructured scaffold, cells align with each other, and bundle-like myofiber organization is observed, where nuclei are distributed alongside the central axis of the fibers, which benefits myogenesis and muscle differentiation.

### Changes transcriptomic profiles in response to flat and microstructured edible scaffolds: connecting muscle fiber generation with global transcriptome profiles

This study used high-throughput sequencing technology and transcriptomic analysis bioinformatics tools to reveal differences in C2C12 cells transcriptome (*Mus musculus*) seeded on scaffolds produced with flat and microstructured surfaces and plastic as control. An average of 86,067,975 raw reads from control, flat, and microstructured samples (Supplementary Table 1) were obtained from 9 samples, 3 corresponding to each condition, and an average clean read of 83,302,338. All the downstream analysis was based on high-quality clean data. Clean reads were mapped to mouse (*Mus musculus*) reference genome sequence version GRCm39 obtained from Ensembl, obtaining approximately 94.8% of the transcripts aligned to the genome.

**Figure 3:**
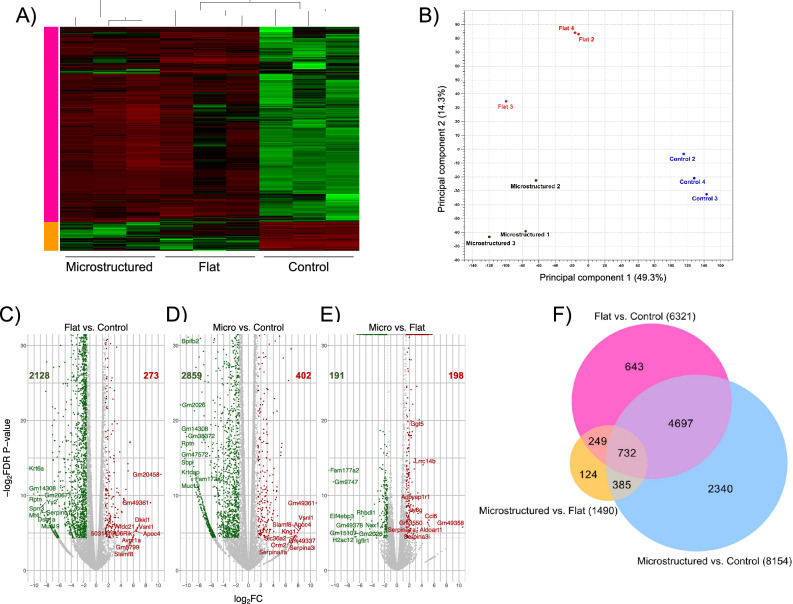
(**A**) Hierarchical clustering analysis for the transcriptome profiles of control, flat, and microstructured samples. The heatmap presents the mean relative abundance of the genes with different colors (green: low abundance; red: high abundance). (**B**) PCA score plot of the control, flat, and microstructured samples. (**C**–**E**) Volcano plots highlighting most differentially expressed genes (Log2FC > 1.5; FDR corrected *p* value < 0.05) for the three comparisons Flat versus Control, Microstructured versus Control, and Microstructured versus Flat, respectively. (**F**) Venn diagram of DE genes per each type of sample. Colored circles represent the number of DE genes for Flat versus Control, Microstructured versus Control, and Microstructured versus Flat respectively comparisons. A total of 6321, 8154, and 1490 DE genes were differentially expressed (FC > 1.5; FDR corrected *p* value < 0.05) for Flat versus Control, Microstructured versus Control, and Microstructured versus Flat respectively.

To assess transcriptome changes within different samples from control, flat, and microstructured samples, hierarchical cluster analysis (HCA) was conducted (Fig. 3A). Samples are displayed as columns and classified by subtypes as indicated by different colors. Control samples showed similar transcriptome distributions and were aggregated into the first cluster. Flat and microstructured samples tended to be clustered together as well. This indicates that using a scaffold, either flat or microstructured, has a clear effect on gene expression, as they aggregate together and differ from gene expression in control samples.

To corroborate the replicates distribution, principal component analysis (PCA) was performed to assess similarity in transcriptome profiles. PCA plots from control, flat, and microstructured replicates (Fig. 3B) showed that the first principal component explained 49.3% of the variability and the second accounted for 14.3% of the total variance. Flat and microstructured can be clearly separated, which indicates replicates consistency.

Global changes in transcriptional profiles are shown as volcano plots (Fig. 3C–E) showing the differentially expressed genes (DEGs), where red indicates genes were significantly upregulated and, green, those significantly downregulated. Figure 3F shows pairwise comparisons between control, flat and microstructured samples. A total of 6321, 8154, and 1490 DEGs were found (|FC|> 1.5; FDR corrected *p* value < 0.05) for Flat versus Control (F vs. C), Microstructured versus Control (M vs. C), and Microstructured versus Flat (M vs. F) comparisons respectively. The amount of DEGs detected in both F versus C and M versus C is much higher than the differences observed between M versus F, where fewer DEGs were found. Interestingly, we found 124 genes expressed exclusively in M versus F associated with proteins that belong to the category of metabolic interconversion enzymes, followed by the categories of cytoskeletal proteins, protein-modifying enzymes, and transporters (Supplementary Table 2). In our overall comparisons, we discovered that genes with the most significant up- and down-regulation have no annotated functions (Table 1). However, the fact that they have significant differences between conditions suggests that these genes could be potentially associated with ongoing cellular processes during muscle fiber development and differentiation on the scaffolds. To further explore the potential functions of this group of genes, we searched the Mouse Genome Database (MGD) and Gene expression Database (GXD) (https://www.informatics.jax.org/). Gene Gm2026, was downregulated in the comparisons M versus C and M versus F, was found to be expressed in limbs, nervous system, and reproductive system. Genes Gm38372 and Gm47572 (downregulated in the comparison M vs. C), were expressed in most of the systems. Finally, Gm14308 is found to be expressed only in the liver and biliary system. To gain additional information, blast searches and domain analyses were carried out to assess their putative molecular functions. For the gene Gm20458, its best blast hit was a SYS1 homolog isoform X3 protein from *Myodes glareolus,* characterized as an integral membrane protein S linking to the trans Golgi network (pfam09801). Gm49361 gene was found to belong to the serine/threonine-protein kinase LATS2 family (isoform X1), providing clues about its potential role in signal transduction during muscle development. In the case of Gm5799, homology searches indicate this gene belongs to takusan superfamily (pfam04822), a large family of uncharacterized proteins. For gene Gm20671, its best blast hit was a PRR14L protein from *Fukomys damarensis* (80% identity and 100% query coverage). Based on domain and motif analyses, Gm20671 encoded protein can be classified as a KRAB box and zinc finger, C2H2 type domain containing protein, potentially involved in transcriptional regulation.

**Table 1:** Top 20 differentially expressed genes (up- and down-regulated) between (A) Flat versus Control, (B) Microstructured versus Control, and (C) Microstructured versus Flat respectively comparisons (|FC|> 1.5; FDR corrected *p* value < 0.05).

Flat versus control			Microstructured versus control			Microstructured versus Flat		
Symbol	Name	Log fold change	Symbol	Name	Log fold change	Symbol	Name	Log fold change
Up-regulated								
Gm20458	Predicted Gene 20458	10.45	Gm49361	Predicted Gene, 49,361	10.92	Gm49358	Predicted Gene, 49,358	10.60
Gm49361	Predicted Gene, 49,361	8.77	Apoc4	Apolipoprotein C-IV	8.36	Ccl6	Chemokine (C–C Motif) Ligand 6	5.16
Wfdc21	WAP Four-Disulfide Core Domain 21	6.64	Vsnl1	Visinin-Like 1	8.25	Aldoart1	Aldolase 1 A, Retrogene 1	4.94
Dkkl1	Dickkopf-Like 1	6.62	Slamf8	SLAM Family Member 8	8.10	Lrrc14b	Leucine Rich Repeat Containing 14B	4.21
Vsnl1	Visinin-Like 1	6.45	Kng1	Kininogen 1	7.88	Ggt5	Gamma-Glutamyltransferase 5	3.59
5031410I06Rik	RIKEN Cdna 5031410I06 Gene	6.45	Gm49337	Predicted Gene, 49,337	7.78	Ly6g	Lymphocyte Antigen 6 Complex, Locus G	3.40
Apoc4	Apolipoprotein C-IV	6.44	Slc36a2	Solute Carrier Family 36 (Proton/Amino Acid Symporter), Member 2	7.66	Adcyap1r1	Adenylate Cyclase Activating Polypeptide 1 Receptor 1	3.36
Slamf8	SLAM Family Member 8	6.06	Orm2	Orosomucoid 2	7.48	Rpl27rt	Ribosomal Protein L29, Retrotransposed	3.31
Gm5799	Predicted Gene 5799	6.06	Serpina1a	Serine (Or Cysteine) Peptidase Inhibitor, Clade A, Member 1A	7.47	Serpina1a	Serine (Or Cysteine) Peptidase Inhibitor, Clade A, Member 1A	3.26
Avpr1a	Arginine Vasopressin Receptor 1A	5.98	Serpina3i	Serine (Or Cysteine) Peptidase Inhibitor, Clade A, Member 3I	7.43	Serpina3i	Serine (Or Cysteine) Peptidase Inhibitor, Clade A, Member 3I	3.24
Down-regulated								
Muc19	Mucin 19	− 8.34	Muc19	Mucin 19	− 9.09	Gm20458	Predicted Gene 20458	− 4.06
Dsg1a	Desmoglein 1 Alpha	− 8.36	Fam177a2	Family With Sequence Similarity 177 Member A2	− 9.16	Zfp273	Zinc Finger Protein 273	− 4.06
Yy2	Yy2 Transcription Factor	− 8.61	Krtdap	Keratinocyte Differentiation Associated Protein	− 9.32	Nox1	NADPH Oxidase 1	− 4.37
Serpinb12	Serine (Or Cysteine) Peptidase Inhibitor, Clade B (Ovalbumin), Member 12	− 8.61	Gm47572	Predicted Gene, 47,572	− 9.42	Rhbdl1	Rhomboid Like 1	− 5.48
Mt4	Metallothionein 4	− 8.72	Bpifb2	BPI Fold Containing Family B, Member 2	− 9.72	Igflr1	IGF-Like Family Receptor 1	− 6.59
Sprr3	Small Proline-Rich Protein 3	− 8.95	Gm38372	Predicted Gene, 38,372	− 9.96	Gm2026	Predicted Gene 2026	− 6.67
Gm20671	Predicted Gene 20671	− 9.13	Gm14308	Predicted Gene 14308	− 10.17	H2ac12	H2A Clustered Histone 12	− 6.76
Gm14308	Predicted Gene 14308	− 9.40	Sbpl	Spermine Binding Protein-Like	− 10.25	Gm49378	Predicted Gene, 49,378	− 6.97
Rptn	Repetin	− 10.05	Rptn	Repetin	− 10.81	Gm15107	Predicted Gene 15107	− 7.00
Krt6a	Keratin 6A	− 11.76	Gm2026	Predicted Gene 2026	− 10.93	Eif4ebp3	Eukaryotic Translation Initiation Factor 4E Binding Protein 3	− 7.47

In Table 1 we can observed that in F versus C comparison, we can highlight among the most up-regulated genes, those that participate in enzyme activation during inflammatory processes (Wfdc21, Log FC: 6.64), co-receptor binding activity (Dkkl1, Log FC: 6.62) and calcium binding activity (Vsnl1, Log FC: 6.45); as for those that are down-regulated we can find genes like Krt6a (Log FC: − 11.76), which codes for keratin, a structural protein part of the cytoskeleton, Rptn (Log FC: − 10.05), which encodes for reptin, an extracellular matrix protein, and Sprr3 (Log FC: − 8.95) which participates in keratinization processes.

In M versus C comparison, some of the most up-regulated genes correspond to Apoc4 (Log FC: 8.36) that participate in lipid transport, Slamf8 (Log FC: 8.10) enables identical protein binding activity, and Kng1 (Log FC: 7.88) that participates in response to stimulus, and the most down-regulated genes are the ones that participate in lipid binding activity (Bpifb2, Log FC: − 9.72), epidermis development (Krtdap, LogFC: − 9.32) and salivary gland development (Muc19, Log FC: − 9.09).

Finally, the most up-regulated genes in M versus F are Ccl6 (Log FC: 5.16), participates in immune system processes, Aldoart1 (Log FC: 4.94) glycolytic process, and Ggt5 (Log FC: 3.59) that has hydrolase activity. As for the genes with the lowest expression, these are Eif4ebp3 (Log FC: − 7.47), which participates in the regulation of translation, H2ac12 (Log FC: − 6.76), which participates in DNA binding, and Igflr1 (Log FC: − 6.59), that participates in protein binding. These high-magnitude changes in the expression of the above-indicated genes provide additional information on the potential downstream responses elicited by the interaction with the flat and microstructured scaffolds.

In addition to these observations, our analysis revealed differential expression of genes with medium and moderated levels within (M vs. F) comparison. Notably, we identified genes responsible for encoding structural components of the extracellular matrix (ECM), such as Elastin (Eln, Log FC: 2.62) and Collagen alpha-1(X) chain (Col10a1, Log FC: 2.52). Additionally, genes encoding sarcomere structural proteins like Myosin, heavy polypeptide 2 (Myh2, Log FC: 2.25), Myosin XVIIIb (Myh18b, Log FC: 2), Obscurin (Obscn, Log FC: 2.17), and Nebulin (Neb, Log FC: 2.02) were identified.

Collagen and elastin are integral to the fibrillar components of the ECM in skeletal muscle, comprising the endomysium, perimysium, and epimysium, which envelop the muscle. The ECM of muscle contributes significantly to the transmission of contractile forces, as well as to developmental processes, growth, and muscle repair^45^.

Genes exhibiting differential expression were also associated with the formation of sarcomeres, encompassing both functional and structural proteins. Apart from myosin, a principal constituent of the sarcomere, known to be key for muscle contraction through its interaction with actin^46^, our analysis also revealed increased expression of genes encoding for nebulin and obscurin proteins. Both nebulin and obscurin play pivotal roles in the structure and function of the sarcomere. Nebulin is involved in regulating actin-myosin interactions, calcium uptake into the sarcoplasmic reticulum, and alignment of Z discs. Obscurin, on the other hand, contributes to myofibrillogenesis for A-band formation, incorporation of myosin, and anchoring the sarcomere to the sarcoplasmic reticulum^47^.

The identification of differential expression in these genes, which encode proteins associated with ECM and sarcomere structural functions, provides insight into the role of microstructured scaffolds in modulating the expression of genes involved in the structure and function of skeletal muscle, particularly when compared to the utilization of flat scaffolds.

### Transcriptional response of muscle fibers response using gene ontology and gene network modeling

**Figure 4:**
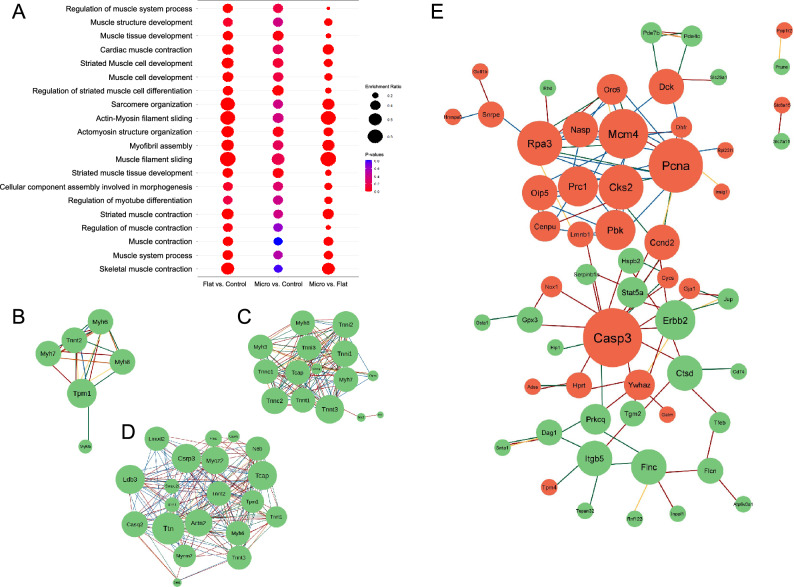
(**A**) GO terms enrichment analysis of differentially expressed genes found in Microstructured versus Flat comparison, on the category of Biological Process. GO terms were considered over-represented using a hypergeometric test to test whether some of the GO terms are over-represented in each gene set, compared to a randomly selected set of genes. Only the first twenty categories were selected based on the *p* value (FDR and Bonferroni corrected) from 4.9 × 10^−10^ to 0. GO term enrichment analysis for Microstructured versus Flat samples from Biological Process category belonging to GO term Muscle filament sliding (**B**), Skeletal muscle contraction (**C**) and Sarcomere organization (**D**). (**E**) Protein interaction network associated with genes found in the Microstructured versus Flat comparison. The nodes with the highest number of interactions are represented in a larger size. Green nodes represent upregulated genes and red ones represent downregulated genes found in Microstructure in comparison to Flat. The colors of the edges correspond to the type of interaction where gray: gene fusion; orange: homology; blue: coexpression; yellow: experimentally determined interaction; green: annotated in databases; red: automated texmining.

A Gene Ontology (GO) term enrichment analysis was performed for each comparison to classify into functions differentially expressed genes. F versus C and M versus C comparisons (Fig. S3) show genes participating in general categories of biological processes, such as translation, cell cycle processes, catabolic processes, and biosynthetic processes, among others. On the other hand, in the analysis performed for M versus F comparison, high enrichment ratio values were obtained, and relevant categories for skeletal muscle development were found (Fig. 4A). The relationship between proteins associated with DEGs of the GO terms for M versus F comparison are illustrated as protein interaction networks generated using String and Cytoscape (Fig. 4B–D). Among the relevant categories found, we can highlight filament sliding, muscle contraction, and sarcomere organization. These results are clear evidence of changes associated with muscle development when the microstructure is present on the scaffold, which was designed to enhance the alignment of the muscle cells. A detailed analysis of each category mentioned above is described as follows.

#### Filament sliding

Muscle cells are composed of myofibrils, which are composed of thin and thick filaments organized axially throughout the cells. Thin filaments contained primarily actin monomers and thick filaments myosin. These filaments are responsible for muscle contraction due to the sliding of them, which results in muscle shortening^48^. Our results show that when compared Microstructured to Flat scaffolds, there are up-regulated genes associated to these filaments, especially myosin heavy (Myh7, Myh6, Mhy8) and light chain (Myl6b) (Fig. 4B), enhancing processes related with filament sliding. Interestingly, the up-regulated gen tropomyosin 1 (Tpm1) shows interaction with all components in the protein network for this category, of which three have been experimentally determined (Myh8, Myh6, and Tnnt2) as informed in String platform.

#### Muscle contraction

Besides actin and myosin, other proteins participate in muscle contraction, such as troponin, titin, and Tcap. All the genes coding for these proteins were up-regulated in microstructured compared to flat scaffold (Fig. 4C). Troponin starts the contraction by binding to Ca^+2^, which results in conformational changes that expose myosin binding sites. At the same time, titin acts as a molecular template that allows the filaments to maintain their length^49^. In the network interaction it is observed that most of the genes interact with each other, except for Stac3, which only interacts with Tnnt3 and Jrsp1.

#### Sarcomere organization

Sarcomeres are the functional unit of the muscle, responsible for force generating and load bearing. Sarcomere structure is organized into different zones: band I, composed of actin; band A, formed by superposition of actin and thick myosin filaments; disk Z, which marks the ends of the sarcomeres; and band M, which marks the center and is composed of myosin. Other proteins such as α actinin, titin, nebulin, and myomesin, are also involved in the structural organization of sarcomeres^46^. Genes associated with all these proteins were found differentially expressed in the M versus F such as Tcap, Lmod2, and Myoz2 (Fig. 4D).

Additionally, a protein interaction network was developed for the 366 DEGs with |FC|> 1.5 and FDR < 0.05 for the comparison of M versus F scaffold (Fig. 4E). It was found that a large part of the negatively regulated genes corresponds to genes associated with processes of regulation of transcription, reproduction, and cell proliferation (Pcna, Mcm4, Rpa3 and, Prc1). Since muscle cells need to exit the cell cycle to initiate the differentiation process^50^, these results suggest that the microstructure enhances the transition process from a proliferative stage to a differentiation stage.

## Conclusion

The proposed microstructured scaffold model designed for cultured meat production achieves precise cell alignment, facilitating the differentiation of myoblasts into cohesive bundle-like formations characterized by evenly distributed nuclei along the myofibers. This configuration effectively curtails the emergence of myofiber branching.

Through comparative transcriptomic analysis, distinct patterns and varying magnitudes emerge within gene expression profiles when comparing F versus C and M versus C, as opposed to the M versus F comparison. This suggests that a smaller subset of genes plays a role in the cellular response to the microstructural cues. Genes that are exclusively up-regulated in the M versus F comparison pertain to crucial aspects of muscle development and differentiation, encompassing functions like the precise organization of sarcomeres, skeletal muscle contraction, and the intricate assembly of myofibrils.

Moreover, a multitude of down-regulated genes are linked to processes that negatively regulate cell division, reproduction, and proliferation. This implies a potential inhibition of cell proliferation in the early stages of differentiation within the sample.

These findings contribute to a deeper comprehension of the intricate interplay between physical stimuli—such as microstructural attributes—and the resulting gene expression patterns and subsequent cellular advancements. Nonetheless, further extensive investigation is imperative to unravel the intricate relationship between gene expression and the impacts of topographical stimuli on cellular morphology.

### Supplementary Information

Supplementary Information.

## Data Availability

All data analyzed during this study are included in this published article. The RNAseq datasets from this study have been deposited in GenBank under accession numbers SRR21631080-91.

## References

[1] Ritchie, H., & M, Roser. Meat and dairy production. In *Our World in Data* 1–35, https://ourworldindata.org/meat-production (2017).

[2] Gerber PJ (2013). Tackling Climate Change Through Livestock: A Global Assessment of Emissions and Mitigation Opportunities.

[3] Bonnedahl KJ, Heikkurinen P (2018). Strongly Sustainable Societies: Organising Human Activities on a Hot and Full Earth.

[4] Bhat ZF, Kumar S, Fayaz H (2015). In vitro meat production: Challenges and benefits over conventional meat production. J. Integr. Agric..

[5] Sharma S, Thind SS, Kaur A (2015). In vitro meat production system: Why and how?. J. Food Sci. Technol..

[6] Freeman CP, Bekoff M, Bexell S (2011). Giving voice to the voiceless: Incorporating nonhuman animal perspectives as journalistic sources. J. Stud..

[7] Potts, A. What is Meat Culture? In *Meat Culture* (Brill, 2016). 10.1163/9789004325852_002.

[8] Weele C, van der Feindt P, Jan van der Goot A, van Mierlo B, van Boekel M (2019). Meat alternatives: An integrative comparison. Trends Food Sci. Technol..

[9] Singh A, Sit N (2022). Meat analogues: Types, methods of production and their effect on attributes of developed meat analogues. Food Bioprocess Technol..

[10] Lee SY (2023). Studies on meat alternatives with a focus on structuring technologies. Food Bioprocess Technol..

[11] Moritz MSM, Verbruggen SEL, Post MJ (2015). Alternatives for large-scale production of cultured beef: A review. J. Integr. Agric..

[12] Simsa R, Yuen J, Stout A, Rubio N, Fogelstrand P, Kaplan DL (2019). Extracellular heme proteins influence bovine myosatellite cell proliferation and the color of cell-based meat. Foods (Basel, Switzerland)..

[13] Datar I, Betti M (2010). Possibilities for an in vitro meat production system. Innov. Food Sci. Emerg. Technol..

[14] Enrione J (2017). Edible scaffolds based on non-mammalian biopolymers for myoblast growth. Materials..

[15] Acevedo CA (2018). Micropatterning technology to design an edible film for in-vitro meat production. Food Bioprocess Technol..

[16] Bezjak D, Orellana N, Valdés JH, Corrales T, Acevedo CA (2023). Towards understanding the role of microstructured edible scaffolds for cultured meat production. Food Bioprocess Technol..

[17] Xiang N (2022). Edible films for cultivated meat production. Biomaterials..

[18] Zhu H (2022). Production of cultured meat from pig muscle stem cells. Biomaterials..

[19] Enrione J (2018). A novel biomaterial based on salmon-gelatin and its in-vivo evaluation as sterile wound-dressing. Mater. Lett..

[20] Acevedo CA (2019). Re-epithelialization appraisal of skin wound in a porcine model using a salmon-gelatin based biomaterial as wound dressing. Pharmaceutics..

[21] Al-Nimry S, Dayah AA, Hasan I, Daghmash R (2021). Cosmetic, biomedical and pharmaceutical applications of fish gelatin/hydrolysates. Mar. Drugs.

[22] Enrione J (2020). Rheological and structural study of salmon gelatin with controlled molecular weight. Polymers..

[23] Bishnoi S (2022). Adjustable polysaccharides-proteins films made of aqueous wheat proteins and alginate solutions. Food Chem..

[24] Ghasemzadeh H, Afraz S, Moradi M, Hassanpour S (2021). Antimicrobial chitosan-agarose full polysaccharide silver nanocomposite films. Int. J. Biol. Macromol..

[25] Gao F, Xie W, Zhang H, Li S, Li T (2022). Molecular mechanisms of browning process encountered in morels (*Morchella sextelata*) during storage. Food Bioprocess Technol..

[26] He H, Liu X (2013). Characterization of transcriptional complexity during longissimus muscle development in bovines using high-throughput sequencing. PLoS One..

[27] Messmer T (2022). A serum-free media formulation for cultured meat production supports bovine satellite cell differentiation in the absence of serum starvation. Nat. Food..

[28] Leal-Gutiérrez J, Elzo M, Carr C, Mateescu R (2020). RNA-seq analysis identifies cytoskeletal structural genes and pathways for meat quality in beef. PLoS One..

[29] Yu Q (2019). Comparative transcriptomics to reveal muscle-specific molecular differences in the early postmortem of Chinese Jinjiang yellow cattle. Food Chem..

[30] Denes LT (2019). Culturing C2C12 myotubes on micromolded gelatin hydrogels accelerates myotube maturation. Skelet. Muscle..

[31] Messmer T (2023). Single-cell analysis of bovine muscle-derived cell types for cultured meat production. Front. Nutr..

[32] Orellana N (2020). A new edible film to produce in vitro meat. Foods..

[33] Jaques A, Sánchez E, Orellana N, Enrione J, Acevedo CA (2021). Modelling the growth of in-vitro meat on microstructured edible films. J. Food Eng..

[34] Bolger AM, Lohse M, Usadel B (2014). Trimmomatic: A flexible trimmer for Illumina sequence data. Bioinformatics (Oxford, England)..

[35] Li R (2009). SOAP2: An improved ultrafast tool for short read alignment. Bioinformatics (Oxford, England)..

[36] Robinson MD, McCarthy DJ, Smyth GK (2010). edgeR: A Bioconductor package for differential expression analysis of digital gene expression data. Bioinformatics (Oxford, England)..

[37] Mortazavi A, Williams BA, McCue K, Schaeffer L, Wold B (2008). Mapping and quantifying mammalian transcriptomes by RNA-Seq. Nat. Methods..

[38] Audic S, Claverie JM (1997). The significance of digital gene expression profiles. Genome Res..

[39] Ashburner M (2000). Gene ontology: tool for the unification of biology. Nat. Genet..

[40] The Gene Ontology Consortium (2023). The Gene Ontology knowledgebase in 2023. Genetics..

[41] Carbon S (2009). AmiGO: Online access to ontology and annotation data. Bioinformatics (Oxford, England)..

[42] Liu B (2011). Analysis of transcriptome differences between resistant and susceptible strains of the citrus red mite *Panonychus citri* (Acari: Tetranychidae). PLoS One.

[43] Rochlin K, Yu S, Roy S, Baylies MK (2010). Myoblast fusion: When it takes more to make one. Dev. Biol..

[44] Listrat A (2015). How muscle structure and composition determine meat quality. Prod. Animales..

[45] Csapo R, Gumpenberger M, Wessner B (2020). Skeletal muscle extracellular matrix—What do we know about its composition, regulation, and physiological roles? A narrative review. Front. Physiol..

[46] Wang Z (2021). The molecular basis for sarcomere organization in vertebrate skeletal muscle. Cell..

[47] Henderson CA, Gomez CG, Novak SM, Mi-Mi L, Gregorio CC (2017). Overview of the muscle cytoskeleton. Compr. Physiol..

[48] Powers JD, Malingen SA, Regnier M, Daniel TL (2021). The sliding filament theory since Andrew Huxley: Multiscale and multidisciplinary muscle research. Annu. Rev. Biophys..

[49] Mukund K, Subramaniam S (2020). Skeletal muscle: A review of molecular structure and function, in health and disease. Wiley Interdiscip. Rev. Syst. Biol. Med..

[50] Kitzmann M, Fernandez A (2001). Crosstalk between cell cycle regulators and the myogenic factor MyoD in skeletal myoblasts. Cell. Mol. Life Sci..

