# Supplementary material for: Global transcriptome profiles provide insights into muscle cell development and differentiation on microstructured marine biopolymer scaffolds for cultured meat production
Source: Sci Rep. 2024 May 13;14:10931. doi: 10.1038/s41598-024-61458-9 (PMC11091069; doi:10.1038/s41598-024-61458-9)
Supplement: Supplementary file 1 — Supplementary Information. [file 41598_2024_61458_MOESM1_ESM.docx]

**Supplementary table and figure legends**

**
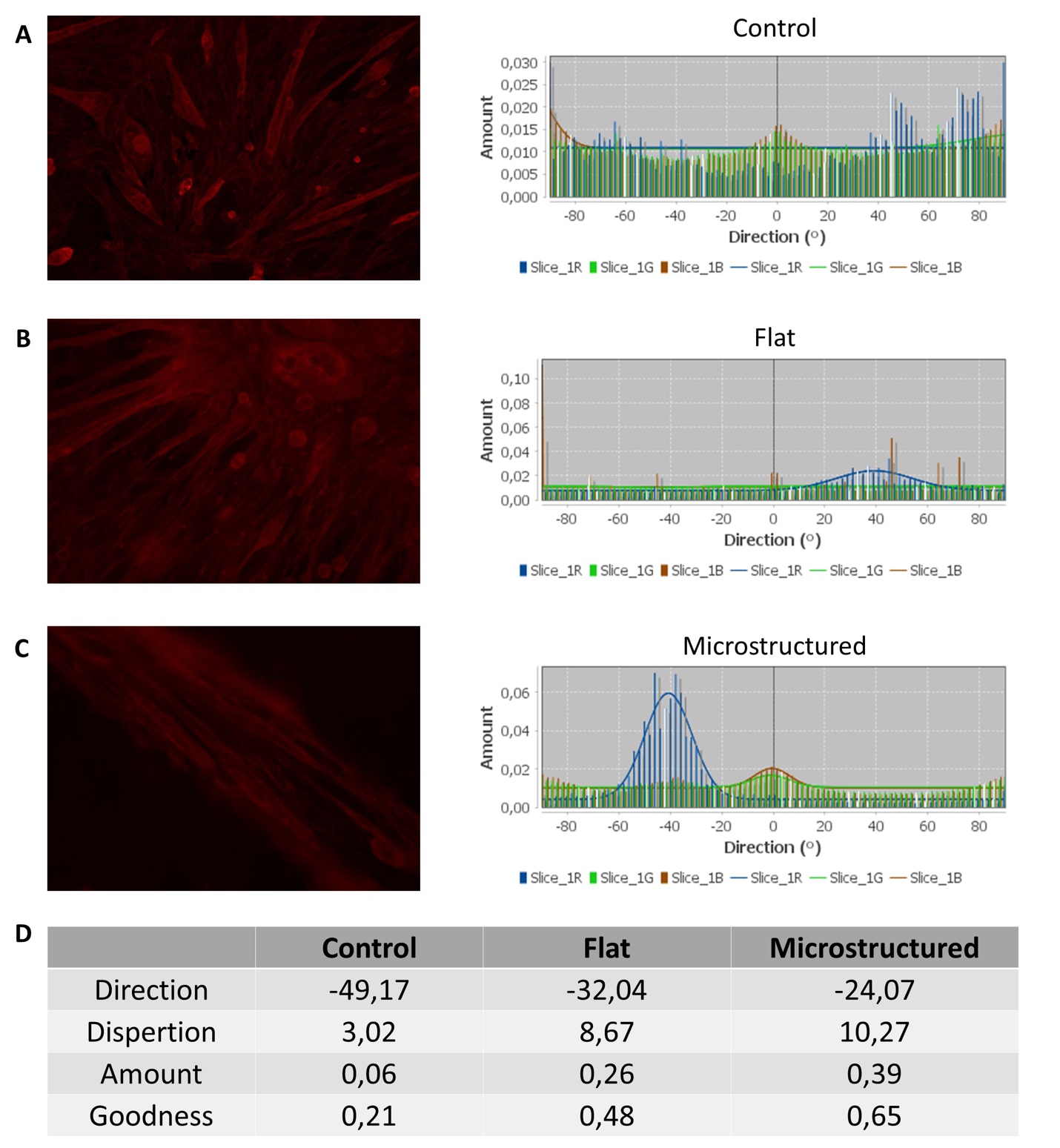
**

**Supplementary Figure 1:** Directionality analysis for (A) Control, (B) Flat, (C) Microstructured and (D) parameters evaluated for directionality.

**Supplementary Figure 2:** Myofiber diameter measurements. Table (A) and plot (B) with values for myofiber diameters.

**
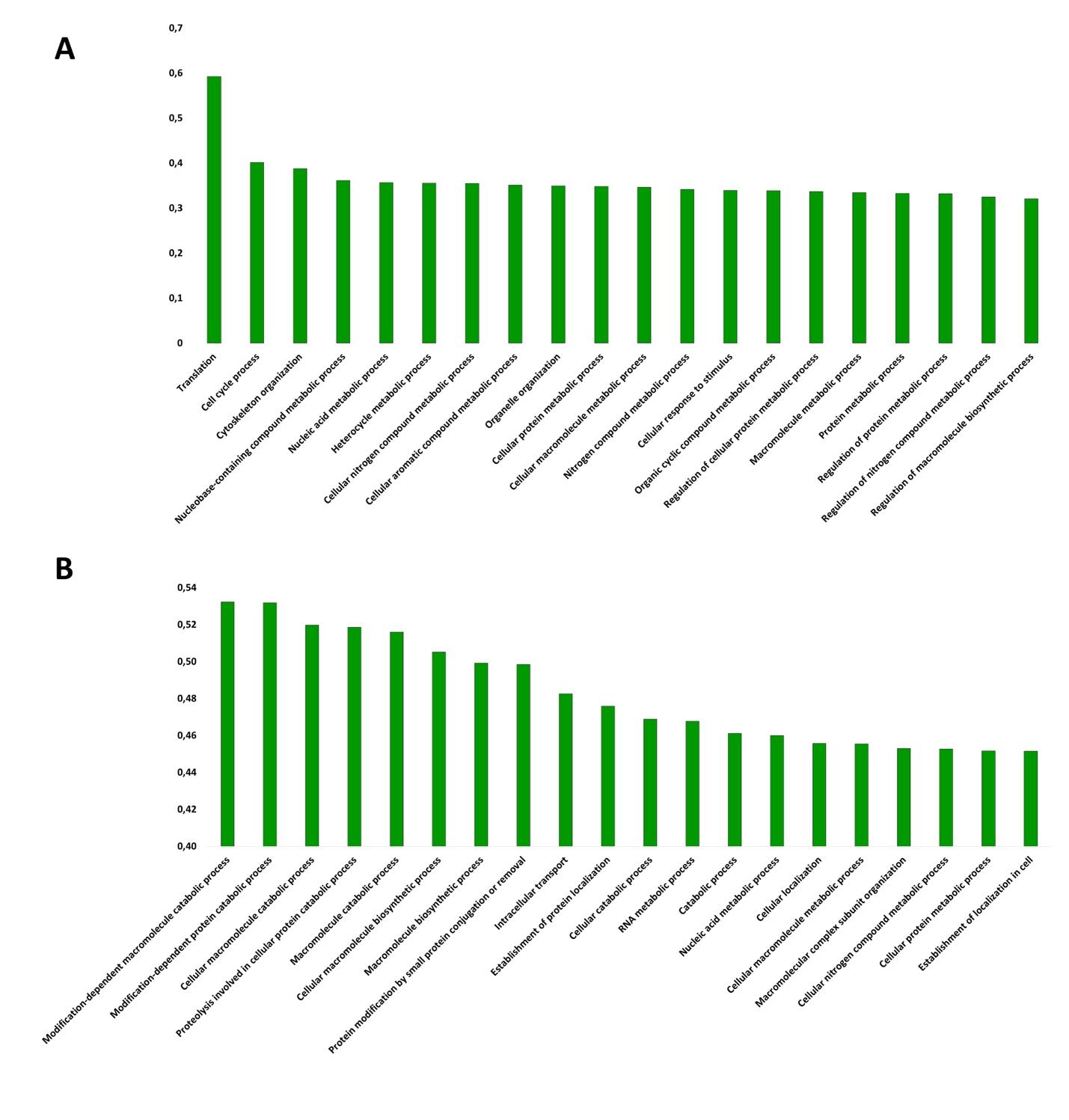
**

**Supplementary Figure 3:** GO terms enrichment analysis of differentially expressed genes in the category of Biological Process. (A) Flat vs Control. (B) Microstructured vs Control.

**Supplementary Table 1.** Reads mapping summary of control, flat and micro structured surfaces.

| **Group** | **Code** | **Total Bases (bp)** | **Raw reads** | **Clean reads** |
| --- | --- | --- | --- | --- |
| Control | C2 | 6,551,238,586 | 43,385,686 | 41,631,020 |
|  | C3 | 8,716,882,398 | 57,727,698 | 56,965,038 |
|  | C4 | 6,628,800,038 | 43,899,338 | 43,015,412 |
| Flat surface | F2 | 9,108,192,556 | 60,319,156 | 57,289,794 |
|  | F3 | 15,922,210,402 | 105,445,102 | 92,454,178 |
|  | F4 | 16,699,647,794 | 110,593,694 | 109,196,306 |
| Micro structured | M1 | 13,690,497,446 | 90,665,546 | 89,520,814 |
|  | M2 | 28,092,960,194 | 186,046,094 | 184,021,086 |
|  | M3 | 11,555,948,762 | 76,529,462 | 75,627,400 |

**Supplementary Table 2.** Categories of principal genes found exclusively in M vs F comparison.

| Metabolite Interconversion Enzyme | | Cytoskeletal Proteins | Protein Modifying Enzyme | Transporters |
| --- | --- | --- | --- | --- |
| Tgm2 | Extl1 | Emp1 | Sbk1 | Sfxn1 |
| Hprt | Aldoart1 | Hip1 | Phkg1 | Slc5a5 |
| Prune1 | Gm3776 | Stmnd1 | Pbk | Trpv1 |
| Plpp1 | Tgm1 | Tmsb4x | Mmp19 | Abca2 |
| Ebp | Dck | Tcp11l2 | Ctsd | Slc19a2 |
| Nox1 | Golt1b | Rilpl1 | Prkcq | Atp6v0a1 |
| Gatm | Dhfr | Coro7 | Prkci | Slc29a1 |
| B4galnt3 | Gpx3 | Tpm4 | Casp3 | Slc7a11 |
| Pde4c | Naa11 | Prc1 | Ccnb1ip1 | Rhd |
| Cyb5r1 | Pde7b | Arpc5 | Sbk2 | Slc6a15 |
